# COVID-19: mask efficacy is dependent on both fabric and fit

**DOI:** 10.2217/fmb-2020-0292

**Published:** 2020-12-22

**Authors:** Steven Darby, Krishnakumar Chulliyallipalil, Milosz Przyjalgowski, Paddy McGowan, Simon Jeffers, Alan Giltinan, Liam Lewis, Niall Smith, Roy D Sleator

**Affiliations:** ^1^The Centre for Advanced Photonics & Process Analysis, Cork Institute of Technology, Bishopstown, Cork, Ireland; ^2^Department of Biological Sciences, Cork Institute of Technology, Bishopstown, Cork, Ireland; ^3^Blackrock Castle Observatory, Cork Institute of Technology, Bishopstown, Cork, Ireland; ^4^Mechanical Energy System Simulation Optimisation Group, Cork Institute of Technology, Bishopstown, Cork, Ireland

**Keywords:** aerosols, coronaviruses, COVID-19, droplets, face coverings, masks, SARS-CoV-2

## Abstract

**Aim:** Face masks are an important addition to our arsenal in the fight against COVID-19. The aim of this study is to present a novel method of measuring mask performance which can simultaneously assess both fabric penetration and leakage due to poor fit. **Materials & methods:** A synthetic aerosol is introduced into the lung of a medical dummy. A conical laser sheet surrounds the face of the dummy where it illuminates the aerosol emitted during a simulated breath. The system is demonstrated with five mask types. **Conclusions:** The curved laser sheet highlights both penetration through the mask fabric and leakage around the edges of the mask. A large variation in both material penetration and leakage was observed.

## Tweetable abstract

Face masks are an effective means of stemming the spread of COVID-19. However, mask performance varies considerably depending on the fabric from which they are made, and how they fit on the face.

## Graphical abstract

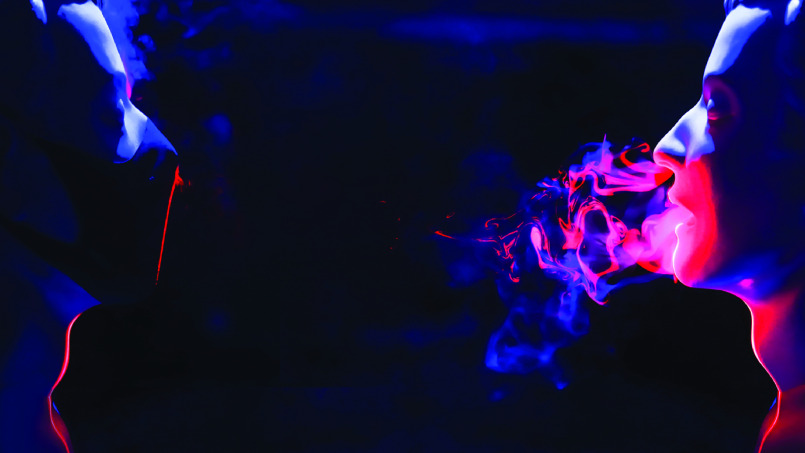

On 26 December 2019, a 41-year-old male was admitted to the Central Hospital of Wuhan, presenting with fever, dizziness and an unproductive cough [1]. The patient, a worker at a seafood market in Wuhan, was one of the first reported cases of a newly emerging severe respiratory disease, which we now know as COVID-19. Metagenomic analysis of a sample of the patient’s bronchoalveolar lavage fluid revealed that the causative agent of COVID-19 is a coronavirus (CoV); named SARS-CoV-2, owing to its phylogenetic relatedness to a group of SARS-like coronaviruses (genus Betacoronavirus, subgenus Sarbecovirus).

SARS-CoV-2 is the seventh member of the Coronaviridae known to infect humans. The first CoV to achieve international notoriety was SARS-CoV; linked to clusters of ‘atypical pneumonia’ (later named SARS; severe acute respiratory syndrome) initially presenting in the Chinese Province of Guangdong, in 2003, before spreading first to Hong Kong and then to some 26 countries; infecting more than 8000 people [2]. While SARS-CoV originated from bats, with human transmission occurring via an intermediate host [3], the true origins of SARS-CoV-2 remain unknown (though bats, pangolins and snakes have all been suggested [4]). As with SARS, COVID-19 spread rapidly from its Chinese epicenter, though with a considerably farther reach and impact. On 11 March 2020, the WHO declared the COVID-19 outbreak a global pandemic [5]. At the time of writing, 216 countries & territories and two international conveyances have reported cases of COVID-19, with the total number approaching 64 million and over 1.4 million associated deaths [6].

Previously we suggested that, in the absence of an effective vaccine, ‘do-it-yourself’ (DIY) face masks were likely to play an important role in stemming the spread of SARS-CoV-2 [7]. The scientific evidence appears to support this, with several reports outlining the role of face coverings in stemming the spread of SARS-CoV-2 [8–13]. However, as the world now faces an inevitable ‘second wave’, it is essential that we redouble our efforts to halt the spread of the virus [14–16]. One approach is to refine and improve face masks, both in terms of fabric and fit.

Face masks worn in a pandemic function more effectively as source control rather than protection [17]. Particles can escape from the mask in two ways- either direct penetration of the fabric, or leakage around the sides [18]. While inward leakage testing is part of the requirements for personal protective equipment (PPE) masks in European Standard (EN) 149, the standard for medical masks, EN 14683, does not have any requirement for a leakage test – neither inward nor outward. The European Committee for Standardisation Workshop Agreement (CWA) 17553 for face coverings also has no quantitative fit test. In fact, to our knowledge, a standard test for outward leakage does not exist. When both leakage and fabric penetration are considered for workplace usage of PPE, the result is the assigned protection factor. These values typically show far lower protection than would be assumed from the performance of the fabric alone. We propose a new metric to take into account outward leakage, which we call outward suppression factor. One would expect outward leakage to be more challenging to fix as the exhaling pressure potentially reduces the seal.

While many recent studies have focused on the penetration of aerosols through various domestic fabrics [19], comparatively fewer studies have looked at outward leakage as opposed to inward leakage. Better fitting medical or N95 masks have been found to significantly improve the source control of particles from a dummy head [17] and better fits have been achieved with an improved mask design, petroleum jelly [20], adhesive tape or fluffed polypropylene fibers [21]. For cloth masks, the only study explicitly addressing fit found a large benefit from wearing a nylon stocking worn over the top of a mask [22], although they measured inward leakage only. Other studies that measured the overall outward mask performance by collecting air around the head did not attempt to separate leakage from fabric penetration as a parameter which contributes to the mask efficiency [23,24]. The extractive techniques used in many of these studies cannot alone distinguish between leakage and penetration. Laser sheets, on the other hand, can visualize the leakage *in situ*. A flat laser sheet is not optimal for this purpose, for example in the study of Fischer *et al.* [25], it is not clear whether leakages would be detected in their laser beam.

Herein we describe, for the first time, the use of a curved laser sheet for simultaneous yet separable detection of both mask leakage and penetration. We envisage this to be used as a tool for mask designers to improve performance without diminishing the breathability of the mask fabric.

## Materials & methods

**Figure 1.**
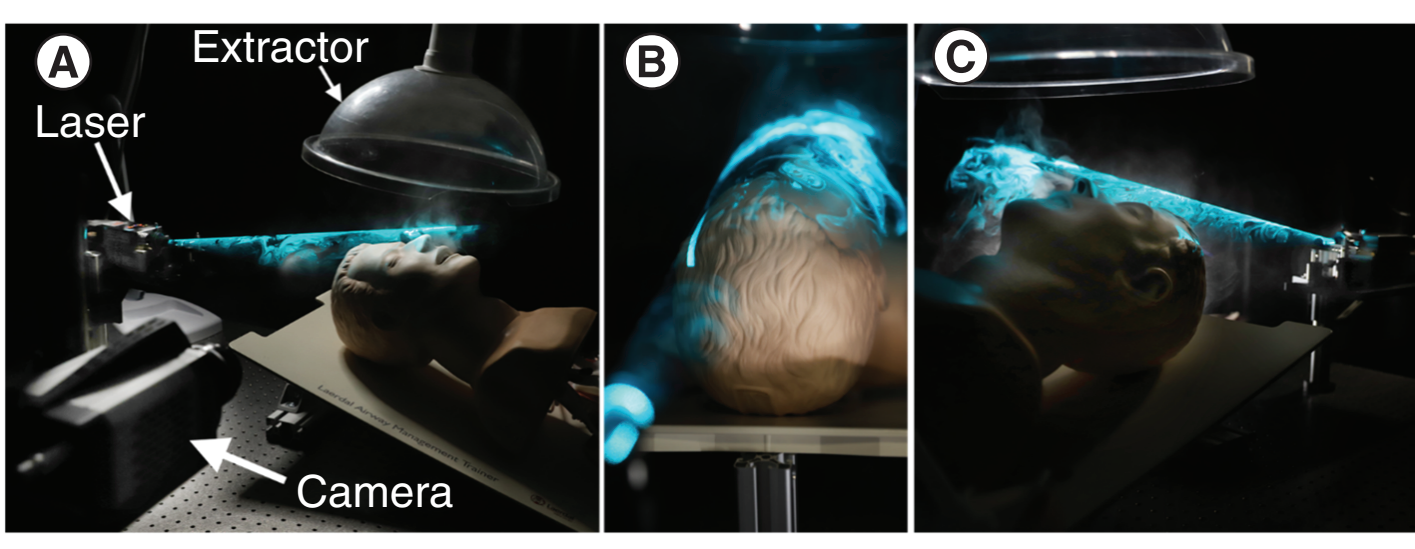
Photographs of the setup, showing the laser cone on an unmasked dummy. The extractor is turned off to enhance laser appearance **(A)** view from the left **(B)** view from top parallel to laser **(C)** view from the right bottom.

The experimental setup is shown in Figure 1. The aerosol consisted of 10% NaCl in water from a nebulizer (Omron NE-C28P, mass median aerodynamic diameter of 3 μm) fed through one of the lung ports of a dummy head (Laerdal airway management trainer) lying face upward on an optical bench. Prior to measurement, the airway was primed with aerosol by turning on the nebulizer briefly while squeezing the attached lung ten-times, then waiting for 30 s for the exhaled aerosol to disperse. A fume extractor was running continuously above the head (ULT Jumbo Filter Trolley 2.0 running at 0.3 m/s in a position 15 cm above the tip of the nose). After initiating the camera acquisition, the lung was squeezed by hand to deliver a breath. These were measured to deliver 300 ml quite reproducibly (wright respirometer). For the breath duration of 1 s, the average flow of 0.3 l/s is similar to the peak flow for a normal breath 0.5 m/s [26]. The aerosol was visualized with a conical laser sheet generated with a 488 nm DPSS laser (Coherent Sapphire 75 mW) and axicon lens (Thorlabs AX1220-A). The conical cyan sheet surrounds the face at a distance of approximately 2.5 cm from the nose and at a closer distance at the cheek to allow space for the masks. A beam block was used at the axicon’s aperture to prevent direct illumination of parts of the dummy.

The camera was a 2048*2048 pixel sCMOS array (Andor Sona 4BV11) with Nikkor 50 mm F1.8 AI MF lens. It recorded a sequence of 30 frames at 7 Hz (acquisition time of 10 ms). Raw 16-bit images were processed in SAOimage DS9 by applying the ‘bb’ colormap on a value range of 100–20,000 counts using log scaling.

**Figure 2.**
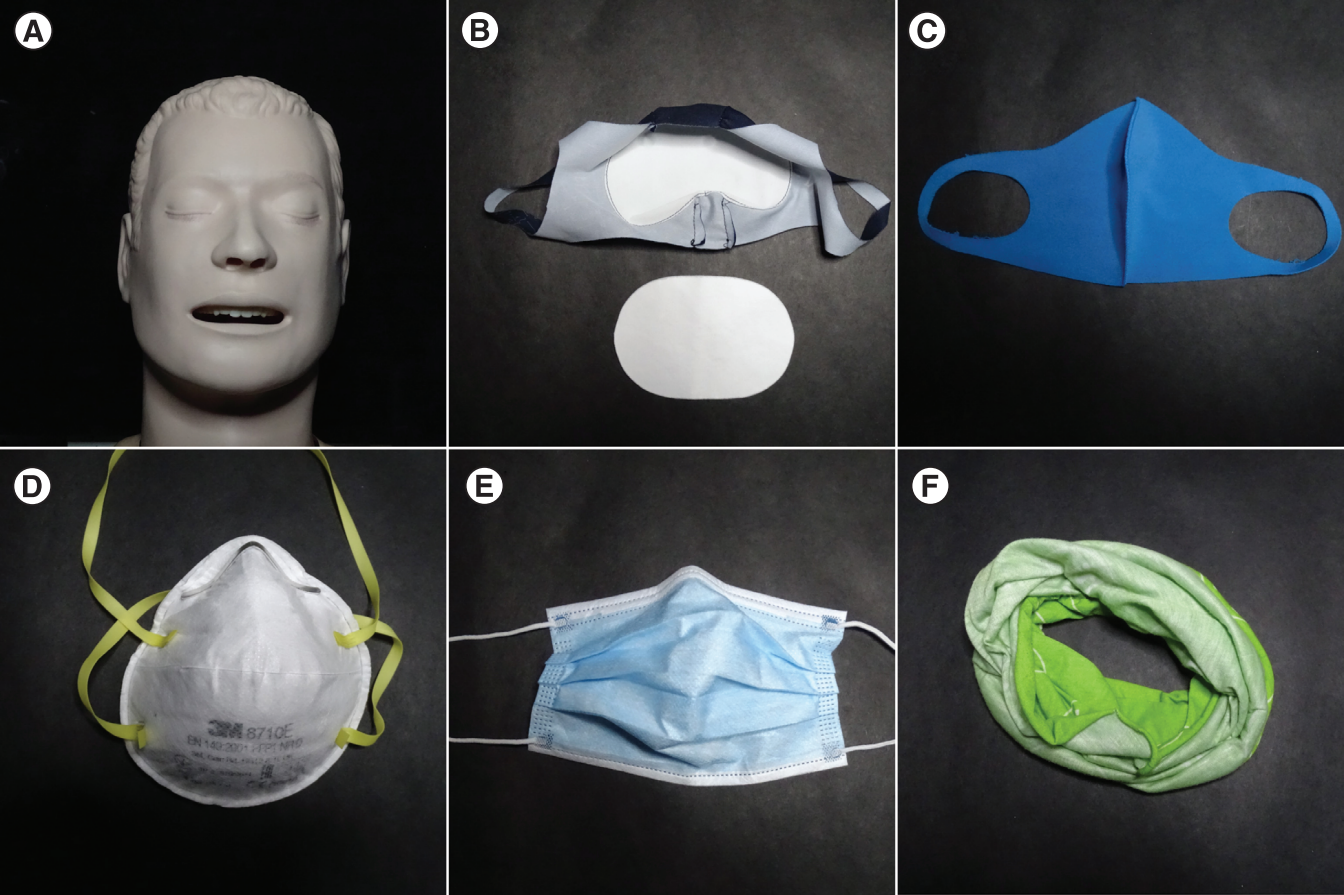
Photographs of the test materials. **(A)** the unmasked dummy, **(B)** three-layer mask with middle filter layer removed, **(C)** single-layer textile mask. Bottom row: **(D)** CE marked FFP1 personal protective equipment mask, **(E)** CE marked Type 2 medical mask, **(F)** bandana.

The masks and dummy used are shown in Figure 2. The three-layer mask in Figure 2B has two outer layers each 0.4-mm thick made of 80% polyester 20% elastane with the internal filter layer being 1.0 mm 100% polypropylene. The filter covers the area of the mouth and nose but around chin, cheeks and nose bridge the fabric is just a single layer. Compliance with the testing methods of the CWA 17553 was claimed by the supplier but packaging was not marked accordingly.

The single layer mask in Figure 2C is of unknown 1.0-mm thick material, of a design which was cheaply and widely available. Compliance with CWA 17553 was not claimed. The CE-marked FFP1 respirator mask in Figure 2D has no exhalation valve and it is marked as complying with EN 149. The CE-marked type 2 medical mask in Figure 2E had a metal nose clip which was cinched down firmly on the dummy. The packaging was marked as complying with EN 14683. The bandana or neck gaiter in Figure 2(f) was 0.33 mm thick 100% polyester and was folded into three layers in these experiments. Dating from 2019 this was never intended for infection control, although the same material has been seen to be used as a face covering.

## Results

**Figure 3.**
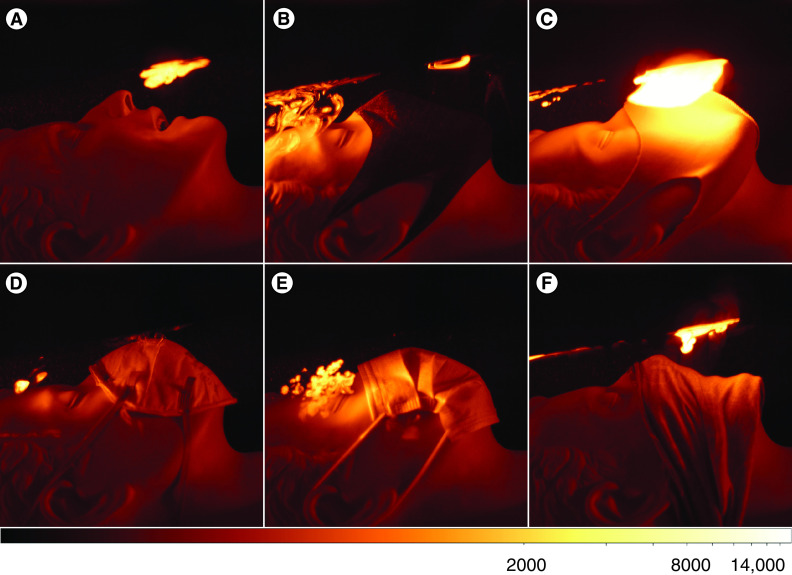
Testing mask fabric and fit. Top row: **(A)** no mask, **(B)** three-layer textile mask, **(C)** single-layer textile mask. Bottom row: **(D)** CE marked FFP1 PPE mask, **(E)** CE marked Type 2 medical mask, **(F)** bandana. Intensity scaled to 100–20,000 counts with ‘bb’ colourmap.

Figure 3A–F shows images of the unmasked dummy and with five different face coverings. The frames shown here are the brightest of the sequence of 30 frames, corresponding to the peak particle emission. All other frames, together with raw data, are available in the supplemental materials. Distinct differences in the effectiveness of the various materials can be clearly seen. The unmasked dummy should have the highest particle emission, but the scattering plume appears relatively dim (Figure 3A). This is because the narrow jet crosses the laser sheet quickly, so that at any one-time there are few particles in the laser sheet. The three-layer mask shows some fabric penetration directly in front of the mouth and some turbulent leakage from under the eyes which crosses the laser cone above the forehead (Figure 3B). The single-layer mask shows very high penetration and some leakage (Figure 3C). The brightness of the penetrating aerosol is around five-times greater than even the unmasked dummy, but this is due to the slower, broader flow which fills the laser beam with aerosol.

Similarly, the bandana shows high penetration (Figure 3F). In this case, the leakage appears less intense, likely as there is less resistance from the fabric that would otherwise force the breath to find a path of lower resistance. The medical mask (Figure 3E) and FFP1 (Figure 3D) mask display no detectable penetration but instead show a few turbulent jets from the cheek and eyes. The leakage at the cheek is easier to discern from inspection of the whole time series (see supplemental materials). In the image of the FFP1 mask, the wisp in front of the mask can be seen to originate at the cheek and did not penetrate the fabric. These leakage jets tend to be directed over the forehead, or across the cheek, and are only partly moved into the laser cone by the extraction air current.

## Discussion

The FFP1 mask is the only one which must pass a fit test to be awarded a CE mark, and indeed it showed the lowest leakage and undetected penetration. For the other mask types, the fabrics with lower penetration showed higher leakage, indicating that these masks displayed a trade-off between fabric and fit.

The scattering of light by small particles is described by Mie theory [27], and is quite non linear. However, using the experimental setup described herein, these nonlinearities will smooth over to a large extent due to the breadth and angle of the laser cone, and wide distribution of particle sizes. The brightness can, therefore, be taken as approximately proportional to the volume of material. Although it is possible to extract pseudo-quantitative results from these images by merely summing up the brightness of each pixel, we caution against doing so for two reasons. As seen in the no-mask image (Figure 3A), a narrow, fast-moving jet appears dim due to the small area of laser sheet it covers, despite carrying a large volume. This weakness could be addressed by more complex image analysis. Another caveat is that the results are dependent on the exact position of the laser sheet. In the configuration used in the current study, for example, any leakage from under the chin would not be detected. However, when using additional laser sheets, it is apparent that almost all leakage occurs around the eyes and cheeks for the masks we tested. These additional laser sheets complicate the interpretation of the images, even if using different laser colors. Directing the cone straight onto the face to form a laser ring around the masks results in a large background scatter from the dummy skin. Although this background can be reduced by black paint and image processing, this configuration would then require another laser sheet to detect penetration.

It is also important to note that the aerosol used in the current experimental setup has a mass median aerodynamic diameter of 3 μm but is also a broad distribution with 10% of the mass in particles larger than 10 μm. This covers the range of particle sizes emitted from speech [28], but whether this also correlates to the infectious particles is not currently known. Most of the fabric testing to date has focused on submicron particles [29,30]. However, a study of influenza in ferrets found the disease was transmitted in particles larger than 1.5 μm, but not smaller particles [31]. For SARS-CoV-2, we do not yet know which size of the aerosol is likely to carry the most infectious viral load. Larger particles are more likely to be captured by the mask material. To an extent this is also true for leaks, as larger particles are more likely to deposit due to impaction when passing around corners.

It is also unknown how different leakage patterns could affect infectivity. Even the worst performing single-layer mask served to slow down the flow from the mouth, which would reduce the spread of particles even if they were not captured. The fate of leakage jets directed to the side, or upward, would depend on the local airflow.

Furthermore, the dummy head used in this study is larger than 95% of adult male heads (tip of the nose to the back of head 237 mm), meaning that the masks were a tighter fit than the average human head. The exact shape of the nose would also have a potentially considerable influence on the leakage. Nonetheless, despite these limitations, our preliminary findings are clear – mask efficacy is dependent on both fabric and fit.

## Conclusion

Given that the reproduction rate of COVID-19 is super-linearly related to both the efficacy of face coverings and the percentage of a population wearing face coverings, a small change in either will result in a large change in disease transmission rates [32]. It is therefore critical that universal public masking will involve optimally suppressive materials that are comfortable to wear for extended periods, can be produced in large quantities from common household materials, and are re-usable and inexpensive. Importantly, such face coverings will not negatively impact PPE supplies [33].

Testing of pandemic masks has been largely focused on the material only. We have demonstrated a technique based on a curved laser sheet that suggests fit is an important parameter in determining mask performance. Measurements like this could help mask designers make improvements simply by altering the design rather than using a different fabric that may potentially be more expensive or have a lower breathability.

Quantitative analysis of the most efficient and effective face masks (in terms of both fit and fabric) will undoubtedly help to stem the spread of not just SAR-CoV-2 but also any illness spread through respiratory particles.

**Summary points**

- The use of face masks has had a significant impact on limiting the spread of COVID-19.
- We provide qualitative evidence based on a novel curved laser sheet which suggests that there is considerable variation in the efficacy of face masks depending not only on fabric type, but also on the fit around the face.
- These results highlight the pressing need for more quantitative analyses based on real life usage to define the most effective face masks for controlling the spread of COVID-19.
- Improved face masks will not only help to suppress the spread of the disease but will also assist in preserving vital personal protective equipment for essential frontline services.
